# Dabigatran - a continuing exemplar case history demonstrating the need for comprehensive models to optimize the utilization of new drugs

**DOI:** 10.3389/fphar.2014.00109

**Published:** 2014-06-10

**Authors:** Brian Godman, Rickard E. Malmström, Eduardo Diogene, Sisira Jayathissa, Stuart McTaggart, Thomas Cars, Samantha Alvarez-Madrazo, Christoph Baumgärtel, Anna Brzezinska, Anna Bucsics, Stephen Campbell, Irene Eriksson, Alexander Finlayson, Jurij Fürst, Kristina Garuoliene, Iñaki Gutiérrez-Ibarluzea, Krystyna Hviding, Harald Herholz, Roberta Joppi, Marija Kalaba, Ott Laius, Kamila Malinowska, Hanne B. Pedersen, Vanda Markovic-Pekovic, Jutta Piessnegger, Gisbert Selke, Catherine Sermet, Susan Spillane, Dominik Tomek, Luka Vončina, Vera Vlahović-Palčevski, Janet Wale, Magdalena Wladysiuk, Menno van Woerkom, Corinne Zara, Lars L. Gustafsson

**Affiliations:** ^1^Division of Clinical Pharmacology, Department of Laboratory Medicine, Karolinska Institutet, Karolinska University Hospital HuddingeStockholm, Sweden; ^2^Medicines Use and Health, Strathclyde Institute of Pharmacy and Biomedical Sciences, University of StrathclydeGlasgow, UK; ^3^Clinical Pharmacology Unit, Department of Medicine, Karolinska Institutet, Karolinska University Hospital SolnaStockholm, Sweden; ^4^Clinical Pharmacology Service, Vall d'Hebron University Hospital, Fundació Institut Català de Farmacologia, Autonomous University of BarcelonaBarcelona, Spain; ^5^Department of Medicine, Hutt Valley DHBLower Hutt, Wellington, New Zealand; ^6^Public Health and Intelligence Business Unit, NHS National Services ScotlandEdinburgh, UK; ^7^Department of Healthcare Development, Public Healthcare Services Committee, Stockholm County CouncilStockholm, Sweden; ^8^Department of Medical Sciences, Uppsala UniversityUppsala, Sweden; ^9^Austrian Medicines and Medical Devices AgencyWien, Austria; ^10^Agency for Health Technology AssessmentWarsaw, Poland; ^11^Department of Finance, Faculty of Business, Economics and Statistics, University of ViennaVienna, Austria; ^12^Hauptverband der Österreichischen SozialversicherungsträgerVienna, Austria; ^13^Centre for Primary Care, Institute of Population Health, University of ManchesterManchester, UK; ^14^Green Templeton College, University of OxfordOxford, UK; ^15^Health Insurance InstituteLjubljana, Slovenia; ^16^Medicines Reimbursement Department, National Health Insurance FundVilnius, Lithuania; ^17^Basque Office for HTA, Ministry of Health and Consumer Affairs, Basque GovernmentBasque Country, Spain; ^18^Norwegian Medicines AgencyOslo, Norway; ^19^Kassenärztliche Vereinigung HessenFrankfurt am Main, Germany; ^20^Pharmaceutical Drug Department, Azienda Sanitaria Locale of VeronaVerona, Italy; ^21^Department of Medicines and Pharmacoeconomics, Republic Institute for Health InsuranceBelgrade, Serbia; ^22^State Agency of MedicinesTartu, Estonia; ^23^Department of Epidemiology and Health Promotion, Public Health SchoolWarsaw, Poland; ^24^Drug Management Department, National Health FundWarsaw, Poland; ^25^Division of Health Systems and Public Health, Health Technologies and Pharmaceuticals, WHO Regional Office for EuropeCopenhagen, Denmark; ^26^Faculty of Medicine, University of Banja LukaBanja Luka, Bosnia and Herzegovina; ^27^Ministry of Health and Social WelfareBanja Luka, Bosnia and Herzegovina; ^28^Wissenschaftliches Institut der AOK (WidO)Berlin, Germany; ^29^IRDESParis, France; ^30^Department of Pharmacology and Therapeutics, Trinity College DublinDublin, Ireland; ^31^Faculty of Pharmacy, Comenius University and Faculty of Medicine, Slovak Medical UniversityBratislava, Slovakia; ^32^Ministry of HealthZagreb, Croatia; ^33^Unit for Clinical Pharmacology, University Hospital RijekaRijeka, Croatia; ^34^Independent Consumer Advocate, VICMelbourne, Australia; ^35^HTA ConsultingCracow, Poland; ^36^Dutch Institute for Rational Use of MedicineUtrecht, Netherlands; ^37^Barcelona Health Region, Catalan Health ServiceBarcelona, Spain

**Keywords:** critical drug evaluation, dabigatran, demand-side measures, managed introduction new medicines, registries

## Abstract

**Background**: There are potential conflicts between authorities and companies to fund new premium priced drugs especially where there are effectiveness, safety and/or budget concerns. Dabigatran, a new oral anticoagulant for the prevention of stroke in patients with non-valvular atrial fibrillation (AF), exemplifies this issue. Whilst new effective treatments are needed, there are issues in the elderly with dabigatran due to variable drug concentrations, no known antidote and dependence on renal elimination. Published studies showed dabigatran to be cost-effective but there are budget concerns given the prevalence of AF. These concerns resulted in extensive activities pre- to post-launch to manage its introduction.

**Objective**: To (i) review authority activities across countries, (ii) use the findings to develop new models to better manage the entry of new drugs, and (iii) review the implications based on post-launch activities.

**Methodology**: (i) Descriptive review and appraisal of activities regarding dabigatran, (ii) development of guidance for key stakeholder groups through an iterative process, (iii) refining guidance following post launch studies.

**Results**: Plethora of activities to manage dabigatran including extensive pre-launch activities, risk sharing arrangements, prescribing restrictions and monitoring of prescribing post launch. Reimbursement has been denied in some countries due to concerns with its budget impact and/or excessive bleeding. Development of a new model and future guidance is proposed to better manage the entry of new drugs, centering on three pillars of pre-, peri-, and post-launch activities. Post-launch activities include increasing use of patient registries to monitor the safety and effectiveness of new drugs in clinical practice.

**Conclusion**: Models for introducing new drugs are essential to optimize their prescribing especially where concerns. Without such models, new drugs may be withdrawn prematurely and/or struggle for funding.

## Background

Pharmaceutical expenditure is under increasing scrutiny, rising by more than 50% in real terms during the past decade among OECD countries (Godman et al., 2013). This rate will continue unless addressed driven by well-known factors including changing demographics and the continual launch of new premium priced products (Garattini et al., 2008; Godman et al., 2013; Malmström et al., 2013). Continued pressure on resources is already resulting in some countries unable to fund new premium priced drugs (Malmström et al., 2013). The number of countries is likely to increase if not addressed with new drugs, including new biological drugs, being launched at US$100,000–400,000 per patient per year or more (Kaiser, 2012; Godman et al., 2013; Malmström et al., 2013). This is in no one's best interest especially if new medicines help improve patients' health either because they are more effective, have less side-effects, are easier to administer than current standards or a combination of these factors (Malmström et al., 2013; Spatz and McGee, 2013).

**Premium prices for new medicines are a concern** among health authorities struggling to maintain, and potentially incompatible with, the European ideals of comprehensive and equitable healthcare (Garattini et al., 2008; Malmström et al., 2013). This can lead to conflicts between authorities and pharmaceutical companies with the latter keen to re-coup the considerable monies spent on Research and Development through encouraging the rapid reimbursement and uptake of new medicines. Uptake can be enhanced by companies spending up to one third of their income on marketing activities alongside lobbying activities (Civaner, 2012; Malmström et al., 2013).

KEY CONCEPT 1 **Concerns with new medicines**

Pharmaceutical expenditure is under increasing scrutiny among health authorities given ongoing pressures. Premium prices for new medicines are a concern especially if there are safety concerns with new medicines in clinical practice, which applied to new oral anticoagulants.

These conflicts between companies and authorities can be greater when there are safety concerns with new drugs, and they are subsequently prescribed in a wider population than studied in randomized clinical trials. Typically Phase III clinical trials are conducted under ideal and highly controlled conditions to seek high internal validity to maximize the chance of demonstrating clinical benefit (Fritz and Cleland, 2003). However, this may lead to substantial differences from their subsequent use in clinical practice. Typically Phase III clinical trials do not include treatment preferences and/or multimodal treatment programs (Wells, 1999; Fritz and Cleland, 2003). Phase III clinical trials may also include a placebo group as a comparator in order to isolate the effects of a particular intervention (Fritz and Cleland, 2003). These situations can lead to concerns with the generalizability of the findings when new drugs are being considered as an alternative to current treatments, especially once prescribed in patients with greater co-morbidities than those enrolled into Phase III clinical trials. They have also led to product withdrawals, which is in no one's best interest. Examples include zimelidine, rofecoxib, and natalizumab (Malmström et al., 2013).

New oral anticoagulants (NOACs) illustrate some of these tensions as they show promise in the prevention of stroke in patients with atrial fibrillation (AF), offering an alternative to warfarin without the need for INR (International Normalized Ratio) monitoring (Pink et al., 2011; Mannuci et al., 2012; Malmström et al., 2013). However, there are safety concerns especially in the elderly in addition to potential compliance problems (Mannuci et al., 2012; Malmström et al., 2013; Marshall et al., 2013; Xu et al., 2013).

AF is the most common clinically significant cardiac arrythmia with an estimated prevalence of 1–2% of the population (Marshall et al., 2013). Current estimates suggest there are 4.5 million people in Europe with AF and 3.03 million in the US (Marshall et al., 2013), with the prevalence of AF likely to double in the next 50 years with ageing populations (Pink et al., 2011; Malmström et al., 2013; Marshall et al., 2013). New drugs are needed since patients with AF have a 5-fold increased risk of cardioembolic stroke compared with those in sinus rhythm (Pink et al., 2011), with a cardioembolic stroke resulting in approximately 20% of patients dying in the acute phase and 60% developing severe disability (Mannuci et al., 2012). In addition, those patients with AF who survive are left more disabled by their stroke and are more likely to have a recurrence than those with other causes of stroke (Malmström et al., 2013).

Anticoagulant therapy with Vitamin K antagonists (VKAs) can reduce by at least 60% the risk of stroke (Malmström et al., 2013). However, there are concerns with warfarin due to the potential of bleeding, the need to tailor doses to the individual with too high a dose potentially causing serious complications and too low a dose losing protection, and the difficulties with maintaining some patients within International Normalized Ratios (Mannuci et al., 2012; Malmström et al., 2013).

Dabigatran received EU marketing authorization in August 2011 (Malmström et al., 2013) for the prevention of stroke and systemic embolism/clot formation in adult patients with non-valvular AF with one or more of the following risk factors:

- Previous stroke, transient ischemic attack or systemic embolism/clot formation.
- Left ventricular ejection fraction < 40%.
- Symptomatic heart failure > New York Heart Association (NYHA) class 2.
- Age > 75 years.
- Age > 65 years in combination with additional vascular risk, i.e., patients with diabetes mellitus, coronary artery disease or arterial hypertension.

Published studies showed a 9% reduction in the prevention of stroke or systemic embolism with dabigatran 110 mg twice daily and 34% for the 150 mg twice daily (Malmström et al., 2013; Marshall et al., 2013). Overall mortality was also reduced by 12% for the highest dose of dabigatran, which reached statistical significance (Mannuci et al., 2012; Malmström et al., 2013). There was also an appreciable and consistent reduction in the risk of haemorrhagic stroke ranging from 69 to 74% depending on the dose of dabigatran. The 150 mg twice daily dose of dabigatran also resulted in a statistical significant reduction in ischemic stroke (24% risk reduction) (Mannuci et al., 2012; Malmström et al., 2013). Dabigatran could also potentially require no monitoring compared with warfarin (Godman et al., 2012; Malmström et al., 2013). As a result, dabigatran has the potential to be an important new treatment, especially where regular monitoring with warfarin is problematic or where there are adverse events or other patient issues with warfarin.

These improvements, coupled with potential savings with dabigatran with the opportunity to reduce patient monitoring, resulted in incremental cost-effectiveness ratios (ICERs) of GB£4831 (€5560)/Quality adjusted life year (QALY) in patients under 80 vs. warfarin and GB£7090 (€8150) above 80 (Malmström et al., 2013). A similar study in Sweden estimated the cost/QALY gained for dabigatran versus warfarin at €7742, increasing to €12,449 in patients who were well controlled with warfarin (Davidson et al., 2013). Other authors have published higher ICERs, e.g., the National Institute for Health and Care Excellence (NICE) increased the base case ICER for dabigatran 150 mg twice to GB£24,173–29,131 (€27,790–33,490)/QALY with different assumptions (NICE, 2012). However, NICE subsequently recommended dabigatran as an alternative to warfarin in patients who meet the criteria outlined in the marketing authorization approval (NICE, 2012).

However, there have been concerns with the rapid introduction of **dabigatran**. There were an appreciable number of serious adverse events within the first 12 weeks of dabigatran's availability in the US (Malmström et al., 2013). These were principally serious bleeding events or blood clots in the elderly (Malmström et al., 2013). Dabigatran was also the most frequently identified medication involving direct safety-related reports to the FDA in 2011. These include haemorrhage, which was the most frequently reported side-effect (Carley et al., 2014). These concerns arose due to dabigatran's low mean oral bioavailability, considerable variation in plasma drug concentrations, and dependence on renal elimination of the active metabolite (Mannuci et al., 2012; Malmström et al., 2013). Consequently, any accumulation of dabigatran in patients with reduced renal function will increase their risk of excessive bleeding, complicated by no effective reversal agent and no commercially available assay to measure blood levels of dabigatran (Legrand et al., 2011; Harper et al., 2012; Mannuci et al., 2012; Malmström et al., 2013; Xu et al., 2013; Reilly et al., 2014). These concerns are enhanced by potentially more elderly patients in clinical practice than seen in the Phase III clinical studies with the belief this would increase the risk of bleeding, which has now been demonstrated (Joppi et al., 2013; Reilly et al., 2014). There were also concerns with dabigantran's budget impact (Malmström et al., 2013). A number of health authorities and health insurance companies across Europe and other countries recognized these issues and initiated extensive pre- and peri-launch programmes to educate physicians and the public regarding the optimal use of dabigatran, especially in elderly patients with poor renal function (Malmström et al., 2013).

KEY CONCEPT 2 **Dabigatran**

The potential for inappropriate prescribing of dabigatran was a concern among health authorities across countries given the considerable variation in plasma concentration levels among the target elderly population, dependency on renal elimination of the active metabolite, no commercially available assay at launch and no effective reversal agent at launch. Consequently, the potential for serious bleeding and deaths if not administered appropriately, which happened in practice.

Consequently, we undertook a review of health authority and health insurance company activities across Europe pre- to post-launch of dabigatran for the prevention of stroke as an exemplar for developing future models to better manage the entry of new premium priced drugs. We subsequently used this knowledge to suggest future activities that all key stakeholder groups could undertake to optimize their use and reduce the likelihood of new drugs being removed from the market place due to safety concerns in a wider patient population. In addition, suggest activities to help control expenditure on new drugs where there are concerns with their budget impact. We have added to this advice based on post-launch studies with dabigatran in a number of countries.

## Methodology

Principally a descriptive review of initial national, regional or local health authority, health insurance company or physician association activities across Europe regarding dabigatran (Malmström et al., 2013).

Demand-side initiatives were collated under four different activities named the 4 Es—Education, Engineering, Economics and Enforcement (Wettermark et al., 2009). Illustrations of these include: (Wettermark et al., 2009; Godman et al., 2013; Malmström et al., 2013):

- **Educational activities**—Ranging from simple distribution of printed material to intensive strategies including academic detailing and monitoring of prescribing habits against agreed guidance usually by professional medical networks.
- **Engineering activities**—Organizational or managerial issues to influence change, e.g., quality and efficiency prescribing targets.
- **Economic interventions**—Financial incentives. These include financial incentives for physicians if they achieve agreed prescribing targets, devolution of drug budgets combined with regular monitoring of prescribing behavior and fines for prescribing costs above agreed limits as well as patient co-payments.
- **Enforcement**—Regulations by law such as compulsory International Non-proprietary Name (INN) prescribing, compulsory generic substitution, or prescribing restrictions such as restricting prescribing of new medicines to a defined patient sub-population.

This has been supplemented by post launch studies evaluating utilization patterns and patient populations in a range of countries and regions. These include Ireland, Italy, New Zealand, Scotland, Slovenia, Spain (Catalonia) and Sweden (Stockholm County Council). Typically, health authority databases and patient registries were used to access relevant patient and utilization data (Malmström et al., 2013).

## Results

### Health authority and health insurance company activities

Table 1 summarizes some of the health authority and health insurance company activities pre-, peri-, and post-launch up till the end of 2012 (Malmström et al., 2013). Unless stated, the indications are those contained in the EMA marketing authorization (Malmström et al., 2013).

**Table 1:** **Summary of key activities across Europe to improve the quality and efficiency of prescribing of dabigatran (Malmström et al., 2013)**.

**Timing**	**Examples of activities among European countries and regions**
Pre-launch (principally education)	Stockholm County Council Systematic and long-term involvement of medical and scientific expertise in the development of guidelines and advice to patients and prescribers through the Regional Drugs and Therapeutic Committee (DTC) and clinical pharmacologists Extensive pre-launch activities with key messages broadcasted both to the public and to prescribers through websites of the DTC as well as the Swedish Medical Journal Appreciable number of pre-launch meetings and training sessions with all major physician groups around the key issues and concerns with dabigatran as well as its likely place in care Production of educational folders regarding dabigatran, slide kits, published articles, and data on the Janus website as well as published information for patients Forecasting the potential budget impact in 2011 and 2012 ahead of launch and monitoring this in practice Development of a laboratory method to monitor dabigatran in plasma with LC-MS/MS technology, and recommending sampling in the introductory phase to build a knowledge database. This to be followed by more situation-based sampling to improve patient safety in the future
Peri-launch (principally education)	(A) Germany Physician Associations stressing when launched that the current knowledge regarding safety with dabigatran was insufficient to answer all questions, and physicians should be careful with prescribing particularly in the elderly The reporting of deaths from excessive bleeding further endorsed these concerns. As a result, limited prescribing in practice in ambulatory care
	(B) Slovenia Reimbursed in conjunction with a complex price: volume agreement
Post-launch (principally education and enforcement)	(A) Austria (education and enforcement) Publication of a guideline “Anticoagulants and Platelet Inhibitors” through a multi-stakeholder initiative including health insurers (Arznei Vernunft, 2014) Ex ex-ante approval by the head physician of the patient's social health insurance fund before reimbursement of dabigatran; otherwise 100% co-payment (mirroring other situations) Renal function has to be assessed and recorded prior to initiation of therapy with dabigatran through determining Creatinine-Clearance (CrCl) levels to exclude patients with severe renal dysfunction (=CrCl <30 ml/min). In addition during treatment, renal function has to be monitored where a decline is envisaged, e.g., patients with hypovolaemia, dehydration and the use of specific additional medication, and renal function has to be assessed at least once a year in patients aged 75 or older, and/or in patients with compromized renal function
	(B) Slovenia Education of all involved specialists and primary physicians on key safety aspects/ adverse events with dabigatran Prescribing restrictions (Enforcement): – Only reimbursed if initiated by an internist or neurologist and prescribed according to agreed indications, e.g., only reimbursed in patients already on warfarin if they are unstable with TTR < 65 – Patients have to be followed in a tertiary or secondary anticoagulation centre. Patients can be followed in primary care but only if authorized by tertiary or secondary center – Every patient has to be registered in a database and followed by the IT anticoagulation programme – Anticoagulation centers have to report once yearly to the tertiary center regarding the number of patients experiencing minor and major bleeding, thromboembolic events, as well as any deaths from bleeding or thromboembolism with dabigatran – No longer a need to report separately to the National Health Insurance (ZZZS)

### Proposed model and associated activities

**Figure 1:**
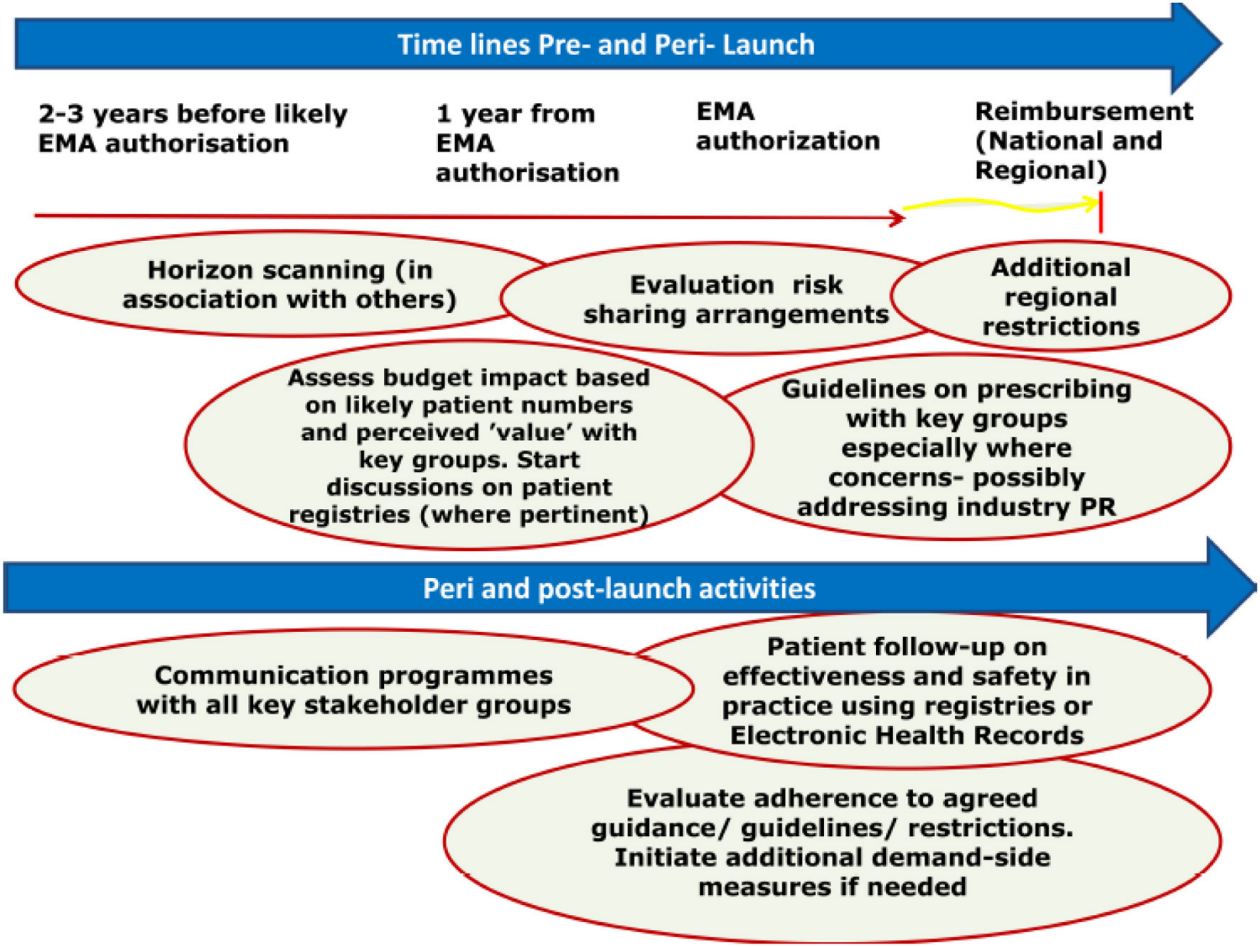
**Proposed model for optimizing the managed entry of new drugs across Europe incorporating national and regional stakeholder groups where pertinent building on the example of dabigatran (Malmström et al., 2013)**.

Figure 1 outlines the suggested new model to **better manage the utilization of new medicines** in the future. This is based on extensive knowledge and experience including pre- to post-launch activities with dabigatran shared across healthcare institutions. This builds on the identified three pillars of pre, peri-, and post-launch activities (Godman et al., 2012).

KEY CONCEPT 3 **Better manage the utilization of new medicines**

Potential models can be developed to optimise the use of new medicines. This starts pre-launch with horizon scanning, budgeting and physician education, through peri-launch with critical evaluation of the potential role and value of new medicines, and carrying onto post launch. Post launch activities include patient registries to monitor the prescribing of new medicines including potential safety issues and outcomes, as well as the prescribing against agreed guidance.

The proposed model starts with horizon scanning activities pre-launch. Post launch activities include monitoring current prescribing, benchmarking and entering patient details on registries.

Key issues for health authorities to consider when appraising possible risk sharing arrangements or managed entry agreements have already been summarized (Malmström et al., 2013). The same applies to key issues and considerations before implementing patient registries (Figure 1).

Overall, there are a number of activities that each key stakeholder group should consider pre-, peri-, and post-launch to better manage the entry of new drugs. This is especially important where there are potential safety and/or resource issues with new premium priced drugs (Malmström et al., 2013).

### Post launch studies

#### Italy

A recent study using a population-oriented database for medicine use aimed to describe clinical features and pharmacological treatments of a population-based cohort of Italian patients discharged from the hospital with a diagnosis of non-valvular AF. Subsequently, to compare these patients with those included in the RE-LY, ROCKET AF, and ARISTOTLE studies for dabigatran, rivaroxaban, and apixaban respectively (Joppi et al., 2013).

Of the 2,862,264 subjects considered for the study, 13,360 patients (0.47%) were discharged from hospitals with a diagnosis of non-valvular AF. Their mean age was 76.3 (SD 10.7), 49.8% were men and 64.6% were ≥75 years of age. This compares with a mean age of 71.5 ± 8.7 in the Re-LY study, a proportion of women < 40%, and approximately 40% ≥75 years of age.

Fifty percent of patients were treated with warfarin and 44.1% with antiplatelet agents. The proportion of patients on antiplatelet therapy increased with age, up to a rate of 54.3% in subjects ≥85 years. 92.9% of the studied cohort were on polypharmacy (mean 8 drugs/patient). Approximately 20% of the entire cohort was treated with amiodarone, a drug potentially interfering with NOACs, and 3.6% from a subgroup analysis had renal failure, which is an exclusion criterion in trials on NOACs (Joppi et al., 2013).

The results of this survey demonstrate once more that considerable efforts should be made by pharmaceutical companies and others to include more women and elderly people in randomized clinical trials. Furthermore, while suggesting a role for NOACs in satisfying patients' needs, there is still unmet need. Further phase IV studies are also required to confirm the benefits and clarify the safety profile of novel oral anticoagulants in routine clinical practice (Malmström et al., 2013).

#### New zealand

A recent study conducted among patients recruited from the medical services at Lower Hutt Hospital and two large general practices in New Zealand also showed less predominance of men than the RE-LY study (42 vs. 63%), an older cohort (73 vs. 71 years) and greater morbidity (Thorne et al., 2014). This is in keeping with differences between patients seen in RCTs compared with those in real life.

The authors did not observe inappropriate prescribing of dabigatran according to patients' renal function. They believed this may reflect extensive local educational initiatives by the Best Practice Advisory Centre (BPAC) as well as initiatives by local prescribers pre- and peri-launch. This suggests that prescribers can be educated to operate within accepted guidelines, providing guidance for the future (Thorne et al., 2014). There were a high number of adverse events reported to New Zealand Centre for Adverse Drug Reaction Monitoring after the launch of dabigatran. However, increased publicity on adverse events and increasing knowledge about dabigatran among prescribers in New Zealand, especially regarding renal function, led to a reduction in number of adverse reactions reported to the New Zealand Centre for Adverse Drug Reaction Monitoring in recent months (Thorne et al., 2014).

#### Ireland

Patterns of dabigatran prescribing were investigated in a national-level prescription claims database. Dispensing records from January-October 2013 inclusive were studied and patients with a dabigatran supply duration of greater than 35 days were assumed to be non-valvular atrial fibrillation patients. 5887 (85%) patients were identified as having received dabigatran, 5012 (83%) of whom received the drug for >35 days. 62% of patients who received dabigatran for >35 days were 75 years or older and 37% were 80 years or older. 56% of patients were male. A high proportion of patients were found to be concurrently receiving drugs which may increase the bleeding risk associated with dabigatran use: 36% of patients receiving dabigatran for >35 days were found to have also received at least one “caution” drug (defined as per SmPC) when dispensed dabigatran.

This analysis followed publications from various authorities in Ireland as there were concerns with the prescribing of dabigatran in clinical practice (National Medicines Information Centre, 2012; Barry, 2013; Irish Medicines Board, 2013). Following the analysis, a letter has now been sent by the authorities to all physicians in Ireland reminding them of the appropriate prescribing of NOACs based on the findings.

#### Scotland

**Figure 2:**
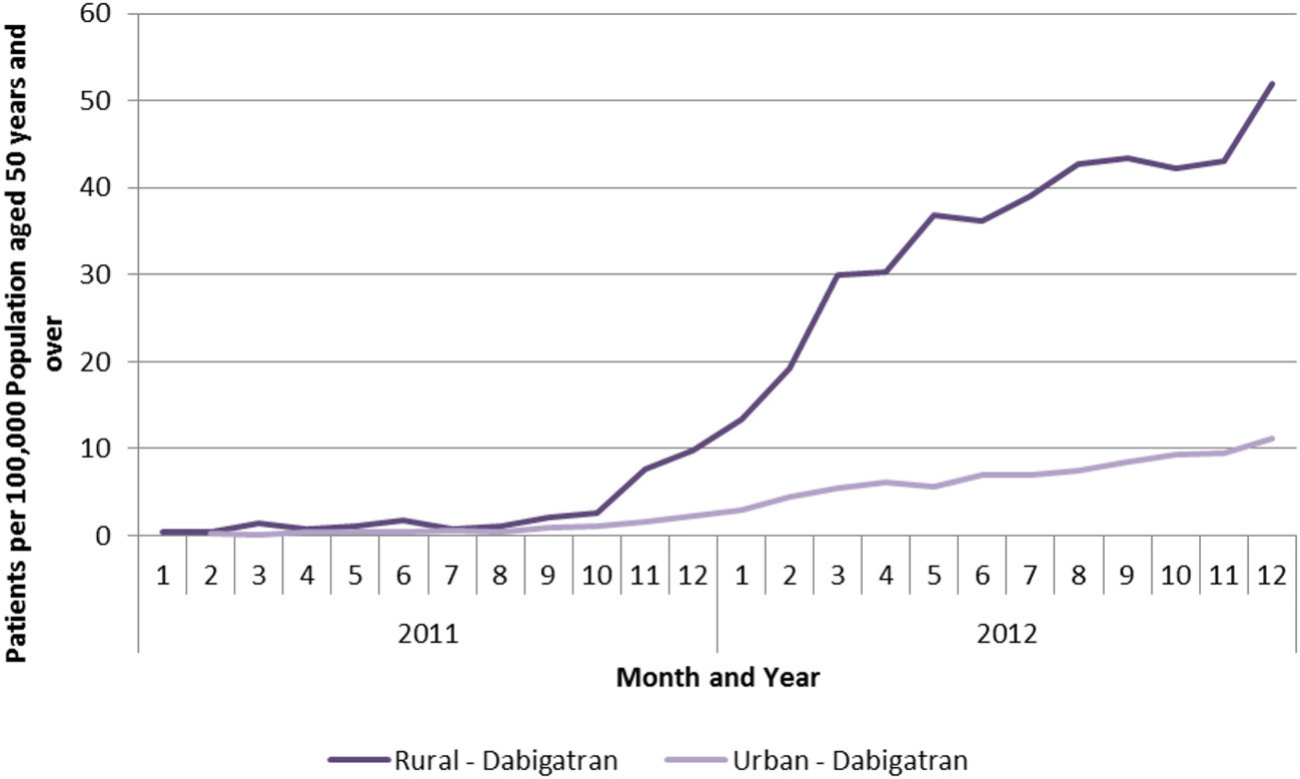
**Patients per 100,000 population aged 50 years and over receiving a prescription for dabigatran by month and type of location (Scott and McTaggart, 2013)**.

In May 2008, the Scottish Medicines Consortium (SMC) accepted dabigatran for use in the prevention of venous thromboembolic events following total hip or total knee replacement surgery (SMC, 2008). However, it was not until late 2011 with acceptance of its use by SMC for the prevention of stroke and systemic embolism in adult patients with non-valvular AF (SMC, 2011) that greater use was seen in ambulatory care (Figure 2) (Scott and McTaggart, 2013). Analysis of the number of patients dispensed a prescription for dabigatran showed a faster and much greater uptake in rural compared with urban areas (Figure 2). The reasons for this are currently being explored, but might reflect differences in access to anticoagulant monitoring services and the perceived advantage of dabigatran in this respect (Malmström et al., 2013). The rates in Figure 2 were standardized against the population aged 50 years or over to take account of different demographics and because this is the age group in which AF is most common.

The overall use of dabigatran, as well as rivaroxaban, still remains modest compared with warfarin in Scotland. This may reflect extensive educational input pre- to post-launch highlighting where pertinent that warfarin should remain first line treatment especially with no known antidote for dabigatran (Malmström et al., 2013).

In contrast to the findings from the other countries described, the population treated with dabigatran in Scotland appears much closer in age and ratio of men to women to those patients included in the RE-LY trial:

- Number of patients: 642 (*M* = 391, *F* = 251).
- Median age: 72 years (*M* = 69, *F* = 75 years).
- Mean age: 70.83 (*M* = 68.92 years, *SD* = 12.4; *F* = 73.81 years, *SD* = 11.599).

This may again reflect a greater use among a broader population in rural areas; however, this remains to be proven. We acknowledge though that we have not broken the ages of patients down by indication. However as seen in Figure 2, there was limited use of dabigatran in ambulatory care prior to SMC approval for its use in patients with AF.

#### Slovenia

Recent analysis of the utilization of dabigatran and warfarin by the Health Insurance Institute of reimbursed prescriptions (Table 2) would suggest that the prescribing of dabigatran is being restricted in accordance to the regulations (Table 1). However, the situation is being monitored in secondary and tertiary anticoagulation centers.

**Table 2:** **Utilization of reimbursed anti-coagulants in Slovenia (DDDs/One Thousand Inhabitants/Day – DID)**.

	**2009**	**2010**	**2011**	**2012**	**2013**
Dabigatran	0.012	0.030	0.058	0.217	1.134
Warfarin	8.553	8.477	8.843	9.329	9.202

There was some utilization of dabigatran prior to reimbursement in August 2012. This may represent use in patients undergoing orthopaedic surgery.

#### Spain (catalonia)

A number of activities were undertaken in Catalonia particularly post launch to help optimize the prescribing of dabigatran (Malmström et al., 2013). A recent follow-up of patients with AF prescribed dabigatran in Catalonia showed the following (Troncoso and Diogène, 2014):

- Patients were older than in the RE-LY study: median age: 77 vs. 71 years and proportion of men lower [52.4 vs. 64.3%] and renal function was lower [creatinine clearance: 25.3% < 50 mL/min vs. 19.4%].
- One-third of the patients followed up were >80 years old; of the 631 patients, 103 were not receiving the recommended dose of dabigatran.
- There were 298 (17.2%) patients with previous ischemic heart disease and 36 (2.1%) patients with severe renal impairment for whom dabigatran should be contraindicated.
- Renal function was not recorded in electronic records during the previous year for 517 patients (30%), and a large number of patients had been prescribed dosages of dabigatran that were not recommended.
- Fifteen patients on dabigatran and oral verapamil were prescribed dosages that are not recommended.

These findings suggest that additional activities are still needed in Catalonia to improve the prescribing of dabigatran. These include information to prescribers, electronic tools to support physicians' decision-making as well as greater monitoring of the prescribing of NOACs (Troncoso and Diogène, 2014).

#### Sweden (stockholm county council)

Analysis of 2363 patients receiving at least one prescription for dabigatran in Stockholm County Council between 2012 and 2013 with a registered diagnosis of non-valvular AF five years or less before the first prescription of dabigatran, and excluding patients where the speciality code for patients' first prescription was surgery/orthopaedics, showed similar ages to patients enrolled into the RE-LY study (Malmström et al., 2013) (Table 3).

**Table 3:** **Patient ages prescribed at least one prescription for dabigatran 2012–2013**.

	***n* =**	**Mean age (years)**	**Min age (years)**	**Max (years)**	***SD***
Female	1002 (42.4%)	73.1	34	96	9.2
Male	1361 (57.6%)	68	24	95	10.7
Total	2363	70.2	24	96	10.4

*NB, Age calculations were based on the patients' age at the first prescription*.

This compares with a recent analysis of 43,353 individuals with non-valvular AF in the Stockholm County Council database between 2006 and2010. The analysis showed 54% of patients were 75 years or older, 39% were 80 years or older, and 44% of patients were women (Forslund et al., 2013).

The strength of dabigatran prescribed varied by age, with lower doses prescribed for more elderly patients:

- 66.9% dispensed 150 mg with a mean age of 66.4.
- 30.1% dispensed 110 mg with a mean age of 77.5 years.
- 3.0% dispensed 75 mg with a mean age of 80.3 years.

Further analysis is planned to see if the lower doses prescribed in patients with a mean ages of 77.5 and 80.3 years correlates with poorer renal function through analyzing their electronic health records.

#### Summary of the findings

The findings from these post launch studies demonstrate that the patient population in clinical practice can be very different to those enrolled in Phase III trials, making extraction from the trial data sometimes difficult. They also demonstrate the need for educating physicians pre-launch if there are safety concerns and contra-indications with new products to reduce adverse drug reactions post launch.

The studies also show the potential to pool the findings from **patient registries** post launch to gain more rapid insights.

KEY CONCEPT 4 **Patient registries**

Patient registries as well as the active monitoring of prescribing of dabigatran were undertaken in a number of countries. These showed the age of the patients in clinical practice was often higher than those seen in the Phase III clinical trials and there was a higher proportion of women. Pre- and peri-launch educational activities among health authorities helped reduce potential adverse drug reactions; however, additional measures are needed in some countries to address dosing concerns and contra-indications to optimise the future prescribing of dabigatran.

## Discussion

Dabigatran and the other new oral anti-coagulants (NOACs) are the result of a long search for an alternative to warfarin to prevent strokes in patients with AF. However, the weighing of the advantages and disadvantages associated with dabigatran, especially in the elderly with poor renal function, needs to be judged carefully and handled appropriately alongside the additional acquisition costs of dabigatran (Malmström et al., 2013; Xu et al., 2013; Carley et al., 2014). This was especially the case in this situation with no widely available assay pre-launch, exacerbated by concerns from the Company regarding the potential for undermining their message about no monitoring (Reilly et al., 2014; Thomas, 2014). These challenges resulted in an extensive range of activities among health authorities, health insurance companies and physician associations across Europe pre- to post-launch to enhance its appropriate use based on factors including age and kidney function (Malmström et al., 2013). Similar findings were seen in New Zealand, Ontario, Canada and Wisconsin, USA (Xu et al., 2013; Carley et al., 2014).

Cases of major bleeding and deaths that were seen with dabigatran soon after its launch, justify the need for the suggested model (Figure 1) to improve the managed entry of new drugs (Legrand et al., 2011; Harper et al., 2012; Mannuci et al., 2012; Malmström et al., 2013). The EMA also reported on 6 November 2011 that there had already been 256 spontaneous reports of serious bleeding resulting in deaths in the EudraVigilance database (EMA, 2011).

Extensive activities among the authorities included educational activities pre-launch in Stockholm County Council, Sweden, as well as post-launch activities among regions and localities in Germany, Spain, Sweden, and the UK (Malmström et al., 2013). There were also prescribing restrictions in some countries alongside the development of shared care protocols between sectors. It is suggested these activities helped reduce subsequent bleeding among patients in practice, especially among those with poor renal function and, as a result, help preserve the availability of dabigatran. However, it is difficult to substantiate this without definite research. Having said this, some of the findings post launch suggests that such activities helped enhance the appropriate use of dabigatran (Carley et al., 2014; Thorne et al., 2014). Further efforts are still needed in some countries to improve the prescribing of dabigatran, e.g., Spain (Catalonia) (Troncoso and Diogène, 2014).

There have also been issues with the additional costs of dabigatran vs. warfarin at GB£919.80 (€1060) per patient per year (UK) given the growing prevalence of AF with currently over 4.5 to 6 million patients across Europe and rising (Malmström et al., 2013). However, there is less of a budget differential in Sweden (Davidson et al., 2013). These combined issues led to (i) prescribing restrictions in some countries alongside prior authorization schemes, e.g., Austria, Belgium, Finland, Slovakia, and Slovenia, (ii) delays with reimbursement in others including Croatia (recently reimbursed as second line to warfarin with a 50% co-payment), the Netherlands, Norway and Portugal (150 mg); and (iii) price: volume and other agreements (risk sharing) to lower the cost of dabigatran, e.g., Ireland, the Netherlands and Slovenia [2]. These concerns also resulted in dabigatran not being reimbursed in some countries, e.g., Estonia, Lithuania, Poland, the Republic of Serbia and the Republic of Srpska (constitutive entity in Bosnia and Herzegovina). Prescribing restrictions and risk sharing arrangements are no doubt preferred by manufacturers versus not having their drugs reimbursed.

The weighing of the benefits and concerns with dabigatran make it increasingly important for European countries and regions to develop and refine models to further improve the managed entry of new premium priced drugs, even if they do not have a tradition of Health Technology Assessment (HTA). As mentioned, the alternative could be reduced resources to fund new drugs in the future, especially with a growing elderly population, which is already happening (Malmström et al., 2013). Such models may also reduce the possibility of new drugs such as dabigatran being withdrawn from the market due to a greater level of side-effects in a wider co-morbid population. None of these alternative scenarios are in the best interests of any key stakeholder group.

We hope we have demonstrated why it is imperative that health authorities and health insurance agencies continue to develop and refine new models to better manage the entry of new drugs. We hope we have also provided direction to all key stakeholder groups to further stimulate the debate about potential activities in this critically important area. This especially as the constant introduction of new premium priced drugs is seen as the greatest challenge to the continued provision of equitable and comprehensive healthcare in Europe (Garattini et al., 2008).

## Conclusion

There were multiple activities pre- to post-launch among authorities across Europe and other countries to improve the prescribing of dabigatran, especially in elderly patients where there are concerns with their renal function. In addition, address potential concerns with the budget impact of dabigatran through for instance price: volume agreements and prescribing restrictions.

We believe and recommend, based on the experiences with dabigatran and other new premium priced drugs, that it is essential that health authorities develop new models to better manage the entry of new drugs in the future (Figure 1). This is becoming critical given the considerable number of new biological drugs in development (Evaluate Pharma, 2012).

Critical activities for health authorities pre-launch include horizon scanning and budget planning activities. This includes identifying products likely to lose their patent within the next one to two years. This is because the price of generics can be as low as 2–10% of pre-patent loss prices, with activities to enhance the prescribing of low cost generics realizing considerable savings (Godman et al., 2013).

Educational materials and clinical guidance also need to be developed pre-launch with the help of physicians and patient groups. Key peri-launch activities include developing prescribing indicators for new treatments as well as the critical appraisal of any proposed risk sharing arrangements. Increasingly discounts are preferred for new drugs rather than complicated arrangements including outcome schemes in view of the complexities involved (Adamski et al., 2010; Ferrario and Kanavos, 2013). Essential post launch activities include monitoring of prescribing against agreed guidance with further educational input if needed. Activities also include increasingly entering patients into registries to monitor the effectiveness and safety of new drugs in wider patient populations.

Without such models, authorities may well struggle to maintain the European ideals of equitable and comprehensive healthcare as well as ensuring funding for new “valued” treatments in target populations. Consequently, the development of new models to better manage the entry of new drugs should be in the interest of all key stakeholder groups.

## Author contributions

Brian Godman, Rickard E. Malmström, Eduardo Diogene, Alexander Finlayson, Magdalena Wladysiuk, Hanne B. Pedersen, Lars L. Gustafsson conceived of the study design, produced the first draft and critiqued subsequent drafts. Christoph Baumgärtel also commented on the differences in design for controlled studies vs. clinical practice. Sisira Jayathissa provided data on New Zealand and critiqued subsequent drafts. Stuart McTaggart and Samantha Alvarez-Madrazo provided data on Scotland and critiqued subsequent drafts. Thomas Cars and Irene Eriksson provided data on Sweden and critiqued successive drafts. Anna Brzezinska and Kamila Malinowska provided input on Poland and critiqued successive drafts. Anna Bucsics and Jutta Piessnegger provided input on Austria and critiqued successive drafts. Stephen Campbell contributed to successive drafts including issues of patient registries and quality indicators. Jurij Fürst provided data on Slovenia and critiqued successive drafts. Kristina Garuoliene provided input on Lithuania and critiqued successive drafts. Iñaki Gutiérrez-Ibarluzea provided input regarding per-launch and post-launch activities including issues such as managed entry agreements and registries. Krystyna Hviding provided input regarding Norway and critiqued successive drafts. Harald Herholz and Gisbert Selke provided input on Germany and critiqued successive drafts. Roberta Joppi provided data on Italy and critiqued successive drafts. Marija Kalaba provided input on Serbia and critiqued successive drafts. Ott Laius provided input on Estonia and critiqued successive draft. Vanda Markovic-Pekovic provided input on the Republic of Srpska and critiqued successive drafts. Catherine Sermet provided input on France and critiqued successive drafts. Susan Spillane provided data on Ireland as well as appropriate references. Dominik Tomek provided input on Slovakia and critiqued successive draft. Luka Vončina and Vera Vlahović-Palčevski provided input on Croatia and critiqued successive drafts. Janet Wale critiqued successive drafts based on her experiences with patients/patient organizations. Menno van Woerkom provided input regarding the Netherlands and critiqued successive drafts. Corinne Zara provided input regarding Spain and critiqued successive drafts.

### Conflict of interest statement

A number of the authors are employed by health authorities or health insurance companies or are advisers to them concerning potential funding of new medicines. Otherwise the authors have no other relevant affiliations or financial involvement with any organization or entity with a financial interest in or financial conflict with the subject matter or materials discussed in the manuscript.
